# Erythraemia

**Published:** 1916-07

**Authors:** 


### ABSTRACTS.

Have you ever felt that this department of the BUFFALO MEDICAL JOURNAL was lacking in abstracts of your specialty, or that such as were published lacked the expert judgement which you yourself could have exercised in selection and preparation? If not, you are more charitable than the editor has been to himself. If so, will you assist in abstracting from a few journals, and thus make this department more representative and more valuable? Incidentally, there are few forms of study more helpful to the worker than this.

**Erythraemia.** G. H. Clark, Glasgow Med. Jour., Edit. in Polyclinic of London, April. Poly(erythro)cythaemia may be due to concentration of blood, increased function of bone marrow as after a haemorrhage, stasis of chronic heart disease and other conditions. Erythraemia was described by Vasquez in 1892 as polycythaemia with cyanosis and may be defined as spenomegalic polycythaemia. Clark's case occurred in a sailor aged 51. Family and personal history negative except for several gastric disturbances several years ago, calling for morphine. He was vaccinated before a trip to Rio de Janeiro and on the trip, red and purple discoloration of the face and neck, less marked on hands, back and sides, was observed. The only subjective symptoms were slight weakness and abdominal fullness. The blood count ran 10-12 millions without casts, and the spleen was enlarged. The urine contained considerable albumin. In May, 1915, swelling of feet and ankles, fullness of upper abdomen, increase of color and thinning of face and arms were observed and the liver was also found to be very large. Blood, July 25, 1915: reds, 10,972,350; whites, 10,230; Hb. 132%; color index 0.605; differential count, Poly. 86.5%, Mononuclears 5.5%, Large lymphocytes 1.75%, Small lymphocytes 2%, Eosinophiles 2.75%, Transitionals 0.25%, Mast cells 1.25%. No poikilocytosis. One normoblast seen. S. G. 1071.83 (normal 1057-1066). By Dreyer's method, the volume of blood was calculated to be 10,118 C.C., about double the normal. Whereas the plasma and corpuscles are normally about equal in volume, here the corpuscles made up 76% of the total. Moisture was calculated at 66%, organic matter and ash at 33.94%. Ash analysis ferric oxid 0.112% (about double the normal); calcium oxid 0.0113% (thrice the normal); magnesium oxid trace; phosphorus pentoxid 0.0126 (fifth of normal); potassium and sodium chlorid and sulphur trioxid 0/854%. After two bleedings, considerable amelioration occurred and some reduction of red cells. References are made to literature but the pathogeny is not understood. Jung's case, quite similar, dying in Jan. 1913, is cited. Here, the reds were 9,000,000 and the whites 30,000, declining to 2,500,000 and 19,000 respectively shortly before death.

